# The Synergistic Effect in CdS/g-C_3_N_4_ Nanoheterojunctions Improves Visible Light Photocatalytic Performance for Hydrogen Evolution Reactions

**DOI:** 10.3390/molecules28176412

**Published:** 2023-09-03

**Authors:** Yu Niu, Jinni Shen, Wenqin Guo, Xiaoyan Zhu, Lanlan Guo, Yueqi Wang, Fuying Li

**Affiliations:** 1 School of Resources & Chemical Engineering, Sanming University, Sanming 365004, China; niuyu200704@163.com (Y.N.);; 2 Department of Engineering Technology Management, International College, Krirk University, Bangkok 10220, Thailand; 3 State Key Laboratory of Photocatalysis on Energy and Environment, Fuzhou University, Fuzhou 350007, China; 4 Fujian Universities Engineering Research Center of Reactive Distillation Technology, Fuzhou University, Fuzhou 350007, China

**Keywords:** CdS/g-C_3_N_4_ nanoheterojunctions, photocatalytic performance, hydrogen evolution reaction, synergistic effect, visible light

## Abstract

This study focuses on the development of heterojunction photocatalysts for the efficient utilization of solar energy to address the energy crisis and reduce environmental pollution. Cadmium sulfide (CdS)/graphite-type carbon nitride (g-C_3_N_4_) nanocomposites were synthesized using a hydrothermal method, and their photoelectrochemical properties and photocatalytic performance for hydrogen evolution reaction (HER) were characterized. Scanning electron microscope images showed the intimate interface and caviar-like nanoheterojunction of the CdS nanoparticles on g-C_3_N_4_ nanospheres, suggesting their potential involvement in the photocatalytic process. Electrochemical and spectroscopic analyses were conducted to confirm the roles of CdS in the nanoheterojunction. The results showed that 10 wt% CdS/g-C_3_N_4_ nanospheres exhibited higher photocatalytic activity than pure g-C_3_N_4_ under visible light irradiation. A HER rate of 655.5 μmol/g/h was achieved after three photocatalytic cycles, signifying good photocatalytic stability. The synergistic effect of the Z-scheme heterojunction formed by g-C_3_N_4_ and CdS was identified as the main factor responsible for the enhanced photocatalytic performance and stability. The interface engineering effect of CdS/g-C_3_N_4_ facilitated the separation of photogenerated electrons and holes. This study provides insights into the design and fabrication of efficient HER photocatalysts.

## 1. Introduction

Photocatalysis has emerged as a promising approach to harness solar energy for various environmental applications, including water splitting, air purification, and wastewater treatment [1,2,3]. In 1972, Fujishima [4,5] and Honda pioneered research on photocatalytic water splitting using TiO_2_ under UV light, which opened up a new avenue for the development of sustainable technologies. Since then, extensive research has been conducted in photocatalysis for environmental applications, with a particular emphasis on degradation and hydrogen evolution reaction (HER). H_2_O photoreduction is a promising approach for addressing the energy crisis and environmental pollution. However, developing efficient catalysts with high performance and good stability remains a significant challenge in this field. To address this issue, researchers have employed various techniques, such as heterojunction formation, surface functionalization, and morphology regulation, to enhance the catalytic activity of HER. The construction of excellent heterojunctions with appropriate cocatalysts is an effective approach for promoting interfacial charge transfer and enhancing the separation of charge carriers [6,7,8,9]. Additionally, the discovery and utilization of various photocatalysts, such as ZnIn_2_S_4_ [10,11], BiVO_3_ [12], metal–organic frameworks (MOF) [13,14], and covalent organic framework (COF) [15,16], has been a major area of research in this field. These materials have shown great promise due to their efficiency and stability, making them attractive alternatives to traditional photocatalysts such as TiO_2_ and ZnO. However, the efficiency of hydrogen production using single-component photocatalysts is severely limited by the rapid recombination of photogenerated charges and the inefficiency of sunlight utilization [17,18]. To overcome these challenges, significant efforts have been directed toward constructing heterojunctions, such as Schottky-type, Z-scheme, type-II, and S-scheme heterojunctions [19,20]. Among these heterojunctions, Z-scheme heterojunctions have received considerable attention due to their unique properties. Z-scheme heterojunctions are designed to achieve a high degree of charge separation and redox ability by recombining the photocarriers that are generated in the absorber layer [21]. Overall, the development of high-performance catalysts with low recombination rates and excellent H_2_ production rates is critical for the practical application of HER. Further research in this area will continue to advance the field and contribute to the sustainable development of renewable energy sources.

Despite the progress made in the development of photocatalysts, several challenges need to be addressed in order to fully realize the potential of this technology for environmental applications [22]. For example, the low stability, limited efficiency, and high cost of some photocatalysts remain significant barriers to their widespread adoption. The selection of a suitable photocatalyst is critical for the success of a photocatalytic process. The discovery of graphite-type carbon nitride (g-C_3_N_4_) [23,24] as a metal-free polymer conjugated semiconductor photocatalyst for H_2_ evolution was first reported by Wang et al. [25]. in 2009. Carbon nitride materials are recognized as promising photocatalysts due to their stability, chemical properties, simple preparation, visible light absorption activity, and low cost. The limited photocatalytic activity of g-C_3_N_4_ can be attributed to three main factors: the easy recombination of photogenerated charges, small sample size, and low specific surface area. To address these issues, many innovative synthesis techniques and modification methods have been proposed to enhance the photocatalytic activity of g-C_3_N_4_. Among these strategies, heterojunction construction has been demonstrated to be effective [26,27].

Cadmium sulfide (CdS) is considered to be an ideal candidate photocatalyst due to its narrow band gap, strong absorption of visible light, and thermodynamic feasibility of proton reduction [28,29]. Moreover, CdS is an attractive material for constructing heterojunctions due to its easily adjustable band structure and high charge separation rate. However, CdS is prone to oxidation into S and Cd^2+^ due to its own photogenerated holes, which limits its application in photocatalysis. Therefore, constructing a novel heterojunction that enables the removal of photogenerated holes from the valence band (VB) of CdS will be crucial for achieving optimal photocatalytic performance [30]. Despite this challenge, many CdS/g-C_3_N_4_ heterojunctions have been reported in the literature, demonstrating the potential of this approach for constructing highly efficient photocatalysts. In addition, the morphology of photocatalysts also plays a crucial role in their photocatalytic performance, with various morphologies such as nanospheres, nanorods, and nanowires reported to enhance photocatalytic performance [31,32]. CdS-C_3_N_4_ heterojunction photocatalysts were synthesized using a deposition method with different masses of CdS on the surface of C_3_N_4_. The mechanism of the photocatalytic reaction was found to follow a photogenerated electron–hole coupling pathway. The high efficiency of CdS-C_3_N_4_ was attributed to its fast photogenerated electron–hole separation capability and good dispersion of CdS on C_3_N_4_ [33]. CdS nanowires were synthesized in situ on a g-C_3_N_4_ nanosheet to generate a heterojunction. This catalyst was used for both P-SOFMR and C-SOFMR, and it was employed for photocatalytic green hydrogen generation. The CN/CdS heterojunction showed excellent photocatalytic activity (6.38 μmol/h in C-SOFMR and 6.16 μmol/h in P-SOFMR at 1.0 mL/min) due to its good optronic properties [34]. In another study, as-formed CdS/g-C_3_N_4_ was reported to have a strong interface due to interlocking binding, necking effect, and heterojunction synthesis, which improved the interfacial separation and transfer kinetics of photogenerated charge carriers. This resulted in a 36-fold increase in the cocatalyst-free hydrogen generation rate compared to pure CdS under visible light irradiation [35].

The CdS/g-C_3_N_4_ semiconductor heterostructure has shown great promise in enhancing the photocatalytic performance of g-C_3_N_4_, primarily by promoting the separation of electron–hole pairs [36]. However, despite its many advantages, CdS/g-C_3_N_4_ still faces some challenges in practical applications [37]. Two major issues are the high cost and the stability of CdS. Therefore, this work aims to study the photocatalytic performances of CdS/g-C_3_N_4_ nanoheterojunctions in the visible light range. The construction of a practical CdS/g-C_3_N_4_ heterojunction photocatalyst with an ideal coupling interface requires a comprehensive understanding of the preparation method to achieve good interfacial charge transfer behavior, a large interfacial contact area, a facile and in situ preparation process, and joint modulation of these three factors. The choice of preparation method can significantly influence the morphology and crystal structure of the CdS/g-C_3_N_4_ interface, which in turn affects the charge transfer process [38]. Tight bonding between CdS and g-C_3_N_4_ is crucial for achieving efficient charge separation and transfer at the interface. A large interfacial contact area is important for promoting efficient charge transfer and separation. The facile and in-suit preparation of CdS/g-C_3_N_4_ is also critical for practical applications. Understanding the mechanism underlying the modulation of these factors is also a critical challenge for optimizing the performance of CdS/g-C_3_N_4_ heterojunction photocatalysts in practical applications.

In this work, g-C_3_N_4_ was synthesized with melamine and potassium chloride as a morphology control template to provide more active sites for photocatalysis. The addition of CdS improved the light absorption properties and inhibited carrier recombination. The photocatalytic HER activity and stability of the prepared CdS/g-C_3_N_4_ heterostructure photocatalyst were also evaluated. A possible mechanism of photocatalytic enhancement was discussed. This study offers a possible route for the synthesis of effective heterostructure photocatalysts.

## 2. Results and Discussion

### 2.1. Crystalline Phases and Texture of Samples

**Figure 1:**
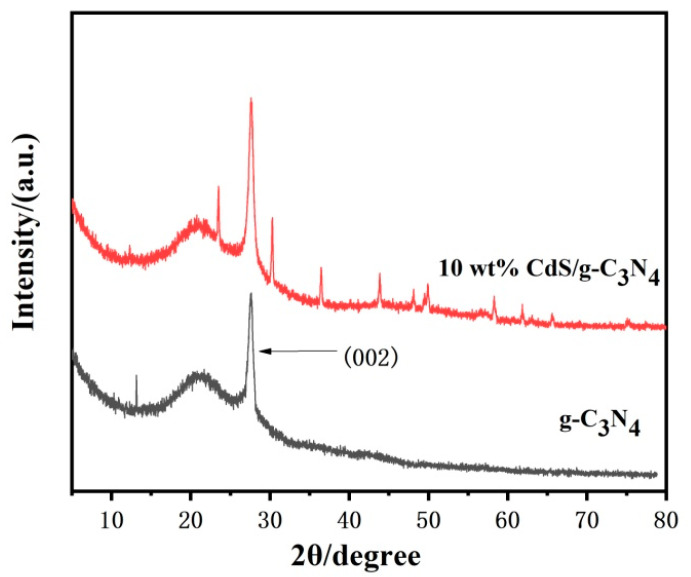
XRD patterns of the samples.

The X-ray diffractometer (XRD) patterns of the pure g-C_3_N_4_ and 10 wt% CdS/g-C_3_N_4_ composites are presented in Figure 1. The strong diffraction g-C_3_N_4_ (002) diffraction peak is attributed to the interlayer stacking of conjugated aromatic systems in the graphite-like carbon nitride. The (100) crystal plane peak is absent due to the weak in-plane repeating structure of the tri-s-triazine ring units [39]. In contrast, the XRD pattern of 10 wt% CdS/g-C_3_N_4_ exhibits strong diffraction peaks at 23.5°, 30.2°, 36.4°, 43.7° corresponding to CdS (JCPDS: 41–1049) [40]. The diffraction peaks of both the g-C_3_N_4_ and CdS phases in CdS/g-C_3_N_4_ provide important information about the crystal structure and phase composition of this material. Previous studies have shown that the diffraction peak intensity of CdS is not significant in 10 wt% CdS/g-C_3_N_4_, which is attributed to the assembly of minor CdS crystallites that are highly dispersed on the g-C_3_N_4_ surface. These XRD patterns indicate that the CdS crystal phase is relatively stable in the prepared composites. This observation is attributed to the strong interaction between the potassium chloride and g-C_3_N_4_ in the synthesized materials, which destroys the interlayer stacking of g-C_3_N_4_ and reduces the crystallinity of g-C_3_N_4_.

### 2.2. Morphologies of Photocatalysts

**Figure 2:**
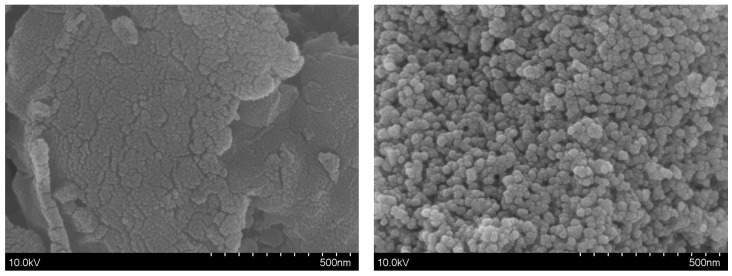
SEM images of pure g-C_3_N_4_ and 10 wt% CdS/g-C_3_N_4_.

The surface morphologies of pure g-C_3_N_4_ and the 10 wt% CdS/g-C_3_N_4_ composite sample were characterized by scanning electron microscope (SEM), as shown in Figure 2. The results show that the lamellar fragmentation of pure g-C_3_N_4_ into granules is different from previous reports. A total of 10 wt% CdS/g-C_3_N_4_ shows uniformly dispersed caviar-like CdS/g-C_3_N_4_ microspheres with diameters of 20–30 nm. Therefore, controlling the degree of crystallization during the preparation of g-C_3_N_4_ plays an important role in the dispersion of CdS.

This SEM analysis demonstrates that small CdS nanoparticles exist on the surface of g-C_3_N_4_ microspheres, which is attributed to the in situ hydrothermal preparation process. These nanoparticles have been suggested to have a significant impact on the interfacial binding and charge transfer properties of photocatalysts, leading to the development of interlocking CdS/g-C_3_N_4_ photocatalysts with enhanced performance. Therefore, an essential condition for the formation of these nanoheterojunction particles is successfully controlling the crystallization of caviar-like CdS/g-C_3_N_4_. However, the exact mechanism underlying the formation of small CdS nanoparticles is still not fully understood, and further research is needed to fully elucidate their role in the formation of nanoheterojunction particles. Controlling the concentration of the precursor solution and adjusting the reaction conditions may be effective strategies for optimizing the size and distribution of these nanoparticles. Overall, the observation of small CdS nanoparticles on the surface of g-C_3_N_4_ microspheres represents an exciting finding with potential applications in the field of photocatalysis.

### 2.3. UV–Vis Diffuse Reflection Spectra

**Figure 3:**
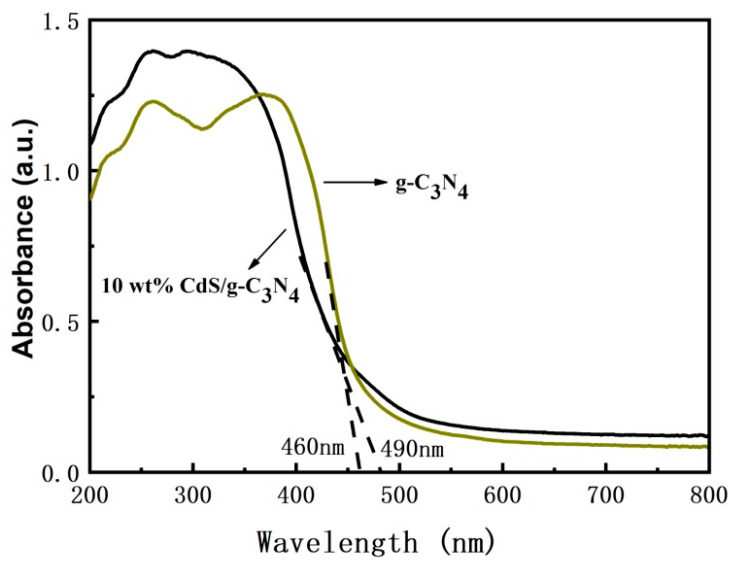
UV–Vis diffuse reflection spectra of the samples.

Figure 3 shows the diffuse reflection spectra (DRS) of the g-C_3_N_4_ and 10 wt% CdS/g-C_3_N_4_ samples. Compared to pure g-C_3_N_4_, CdS/g-C_3_N_4_ composites samples show enhanced absorption in the visible light region. Pure g-C_3_N_4_ exhibits intense light absorption in the entire UV–Vis light spectrum, but the range of light absorption is limited to only about 460 nm. In contrast, the addition of 10 wt% CdS nanoparticles significantly expands the light absorption range of g-C_3_N_4_ from 460 nm to 490 nm. The significantly red-shifted absorption bands are attributed to the type of loaded CdS nanoparticles and the strong absorption of CdS in the UV and visible regions. Thus, more photogenerated electrons and holes are formed in the CdS/g-C_3_N_4_, improving the photocatalytic performance of g-C_3_N_4_.

**Figure 4:**
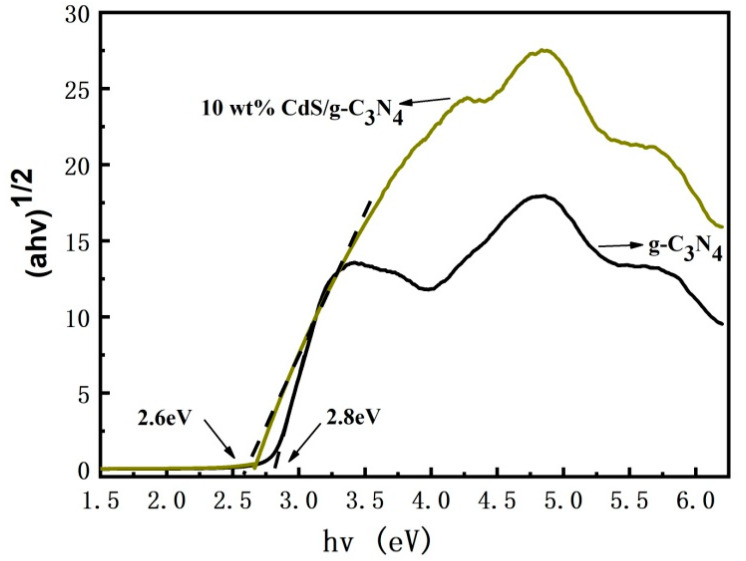
Plot of transformed Kubelka–Munk function versus photon energy of the samples.

Figure 4 shows (ahv)^1/2^ vs. (hv) plots obtained with the Kubelka–Munk function [41], which were used to obtain the energy band gap values of the samples. Pure g-C_3_N_4_ and 10 wt% CdS/g-C_3_N_4_ have band gaps of 2.8 eV and 2.6 eV, respectively. An amount of 10 wt% CdS/g-C_3_N_4_ has a reduced band gap because the band gap of CdS is narrower than that of g-C_3_N_4_. This results in a staggered band arrangement and the formation of a heterojunction system.

### 2.4. Photoelectrochemical Properties and Electrochemical Impedance Spectrum Measurements

**Figure 5:**
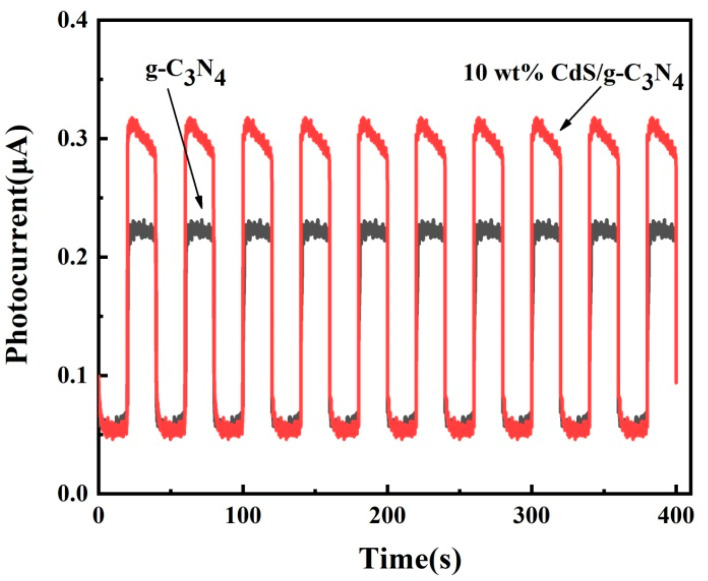
Transient photocurrent response of samples under intermittent visible light irradiation.

The photoelectrochemical properties of g-C_3_N_4_ and CdS/g-C_3_N_4_ were evaluated. Figure 5 shows the transient photocurrent responses of the samples. The g-C_3_N_4_ and CdS/g-C_3_N_4_ samples all exhibit visible light responses. The photocurrent density of 10 wt% CdS/g-C_3_N_4_ is significantly higher than that of g-C_3_N_4_ under 420 nm light irradiation. The higher photocurrent density of 10 wt% CdS/g-C_3_N_4_ reveals the acceleration of charge carrier separation and transfer in this photocatalyst. This result demonstrates that the formation of CdS/g-C_3_N_4_ nanoheterojunctions significantly improves photoexcited charge carrier separation.

**Figure 6:**
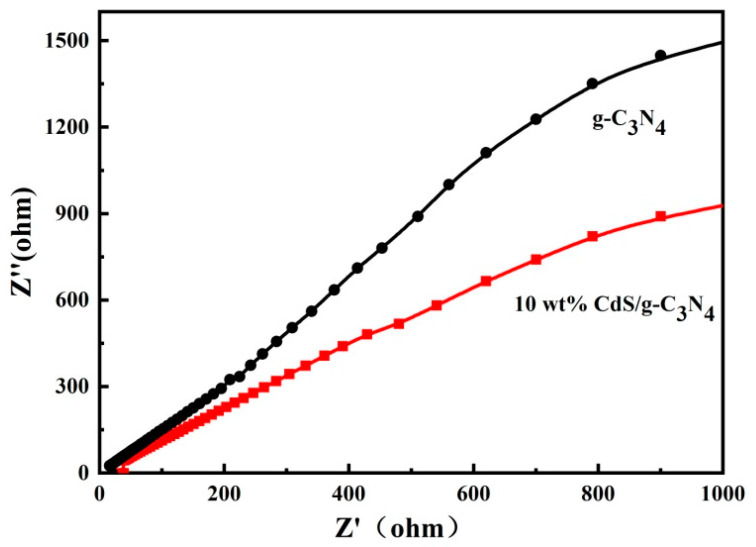
Electrochemical impedance spectroscopy of the samples.

Figure 6 shows the electrochemical impedance spectra (EIS) of the samples. The radius of 10 wt% CdS/g-C_3_N_4_ is smaller than that of g-C_3_N_4_, indicating that nanoheterojunction formation enhances the electron transfer efficiency and increases charge mobility. Therefore, the strong combination of CdS with g-C_3_N_4_ effectively decreases the interfacial charge transfer resistance, which is beneficial for enhancing the separation and transfer of photogenerated charge carriers as well as improving the photocatalytic performance of 10 wt% CdS/g-C_3_N_4_.

### 2.5. Photocatalytic Performance for Hydrogen Evolution Reaction

**Figure 7:**
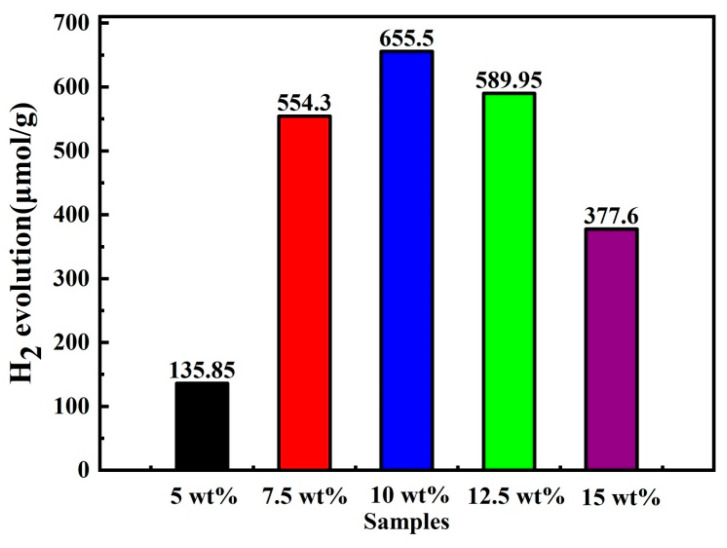
Photocatalytic HER rates of CdS/g-C_3_N_4_.

The photocatalytic activity of the prepared catalysts for hydrogen production was evaluated using online test equipment, and the corresponding results are presented in Figure 7. This reaction was performed under visible light irradiation, which is a more sustainable and cost-effective method compared to using precious metal cocatalysts. The bar chart highlights a comparison of the photocatalytic HER rates of the 5, 7.5, 10, 12.5, and 15 wt% CdS/g-C_3_N_4_ samples. A total of 5 wt% CdS/g-C_3_N_4_ barely produces hydrogen within 5 h, while 7.5 wt% CdS/g-C_3_N_4_ generates 554.3 μmol/g/h of hydrogen during the same period; 10 wt% CdS/g-C_3_N_4_ exhibits the best H_2_ generation rate of 655.5 μmol/g/h, which is 4.8 times higher than that of 5 wt% CdS/g-C_3_N_4_. These results demonstrate that the quantity of CdS plays a crucial role in determining the photocatalytic performance of CdS/g-C_3_N_4_. The improved H_2_ generation rate is attributed to the construction of CdS and g-C_3_N_4_ heterojunction, which enhances charge carrier separation. As the content of CdS increases from 5% to 10%, the photocatalytic activity also increases. However, a further increase in CdS content results in a decrease in the H_2_ generation rate. The HER rates of the 12.5 wt% CdS/g-C_3_N_4_ and 15 wt% CdS/g-C_3_N_4_ photocatalysts are 589.95 μmol/g/h and 377.6 μmol/g/h, respectively.

**Figure 8:**
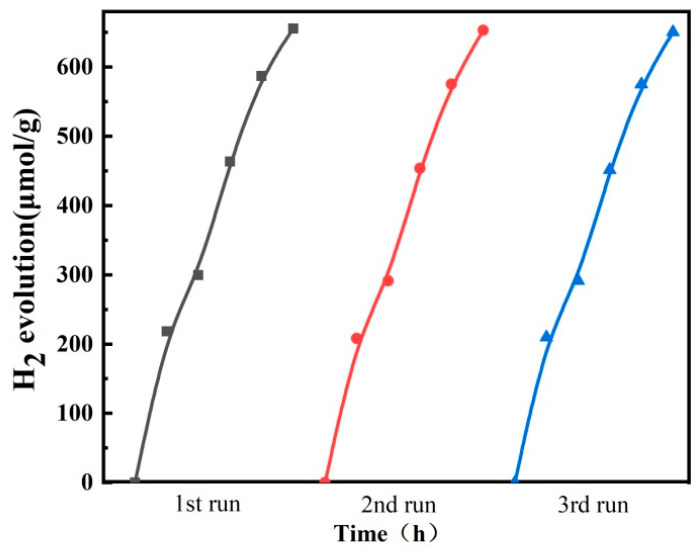
Photocatalytic stability of 10 wt% CdS/g-C_3_N_4_ for photocatalytic HER.

To investigate reusability, the 10 wt% CdS/g-C_3_N_4_ sample was collected and reused three times. Figure 8 displays the results of three successive HER runs under identical conditions. Notably, the 10 wt% CdS/g-C_3_N_4_ sample does not experience a significant loss in photocatalytic activity, and the H_2_ production amount is stable after three cycles. This is due to the feasible transfer of the photogenerated electrons from g-C_3_N_4_ to CdS in the CdS/g-C_3_N_4_ nanoheterojunction, meaning that the photocorrosion caused by the CdS can be effectively avoided. In summary, the in situ hydrothermal preparation method is an effective approach for the preparation of heterostructure photocatalysts. This method involves controlling the crystallization of a caviar-like morphology during photocatalyst synthesis, resulting in the formation of a highly stable and robust structure.

### 2.6. Photocatalytic Hydrogen Evolution Reaction Mechanism

**Figure 9:**
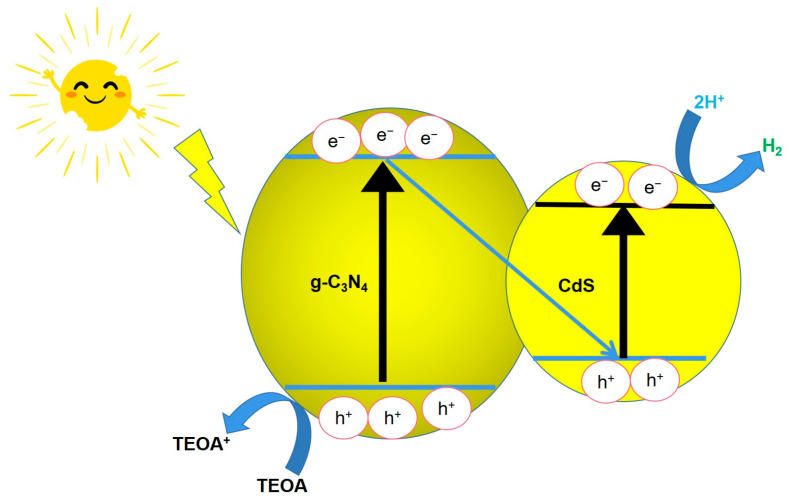
Schematic diagram of charge separation and transfer process.

Based on the experimental results, a proposed mechanism for H_2_ production over the CdS/g-C_3_N_4_ photocatalyst under simulated solar light irradiation is presented in Figure 9. The proposed photocatalytic process involves the excitation of the conduction bands (CB) and valence bands (VB) of CdS and g-C_3_N_4_ by visible light. The staggered band arrangement in CdS/g-C_3_N_4_ facilitates efficient charge separation and migration. The transfer of photogenerated electrons from the CB of g-C_3_N_4_ to the VB of CdS and their recombination with holes rapidly occurs [42]. This results in the accumulation of electrons in the CB of CdS, which exhibits a stronger reduction ability that drives the conversion of H_2_O to H_2_. The photogenerated holes are consumed by the sacrificial agent (triethanolamine (TEOA)), and the oxidation reaction occurs on the surface of g-C_3_N_4_. Clearly, the rate of H_2_ evolution increases with increasing CdS content to a certain extent. This change depends on the transfer of photogenerated electrons from g-C_3_N_4_ to CdS, which reduces the recombination of electrons and holes. The CdS/g-C_3_N_4_ nanojunctions have an enhanced visible light response and adsorption ability compared to pure g-C_3_N_4_, and they possess better anticorrosion properties than CdS. As a result, the CdS/g-C_3_N_4_ composite shows far better photocatalytic HER effectiveness and improved stability compared to pure g-C_3_N_4_. The Z-scheme heterojunction structure of CdS/g-C_3_N_4_ provides a favorable environment for charge separation and transfer, making it an attractive material for photocatalytic applications. The in situ hydrothermal method used in the preparation of CdS/g-C_3_N_4_ provides a facile and efficient way to synthesize the heterojunction structure, which is crucial for the success of the photocatalytic process.

The photocatalytic process proposed in Figure 9 involves several key steps that contribute to the efficiency and stability of the as-formed CdS/g-C_3_N_4_ nanoheterojunction. One important aspect is the tight interface induced by the in situ growth pattern, which ensures a reliable and stable charge transfer pathway between CdS and g-C_3_N_4_. Additionally, the synergistic effect triggered by CdS nanoparticles accelerates the transfer of charge carriers in the Z-scheme heterojunction, leading to improved photocatalytic performance. The accelerated transfer of electrons from g-C_3_N_4_ to CdS further enhances the photocatalytic stability of the as-formed composite photocatalyst. Overall, this proposed process offers a promising strategy for the development of high-performance photocatalysts for water splitting and other related applications [43].

## 3. Experiments

### 3.1. Photocatalyst Preparation

All the chemicals were of reagent grade and used as received without any further purification.

#### 3.1.1. Preparation of g-C_3_N_4_

A certain amount of potassium chloride and 5 g melamine (mass ratio = 10:1) were added to a mortar and ground into a uniform powder. The mixture was roasted at 550 °C for 4 h in a muffle furnace under an air atmosphere at a heating rate of 2.5 °C/min. The obtained powder was washed with deionized water and ethanol three times and centrifuged (7000 rpm/min), and the obtained yellow powder sample was labeled as g-C_3_N_4_ [44].

#### 3.1.2. Preparation of CdS/g-C_3_N_4_

The CdS/g-C_3_N_4_ samples were prepared using a hydrothermal method [45]. A mixture of Cd(CH_3_COO)_2_·2H_2_O and Na_2_S·9H_2_O was dissolved in 60 mL of deionized water. Next, a certain amount of g-C_3_N_4_ powder (CdS and g-C_3_N_4_ mass ratios = 5%, 7.5%, 10%, 12.5%, 15%) was added to the solution. This solution was stirred and sonicated for 0.5 h to achieve a homogeneous mixture. Next, the mixture was transferred to a Teflon-lined autoclave and heated at 180 °C for 5 h. Each obtained powder was washed with deionized water and ethanol three times. The products were dried at 60 °C in air for 12 h and ground for 1 h. The obtained products were denoted as 5 wt% CdS/g-C_3_N_4_, 7.5 wt% CdS/g-C_3_N_4_, 10 wt% CdS/g-C_3_N_4_, 12.5 wt% CdS/g-C_3_N_4_, and 15 wt% CdS/g-C_3_N_4_.

### 3.2. Characterization of Photocatalysts

An X’Pert XRD was used to analyze the phase compositions of the samples. An SEM was used to study the microstructures of the samples. A UV–Vis spectrometer (UV–Vis) was used to record the DRS of the samples. An electrochemical workstation (Epsilon) was used to determine the light/dark short-circuit photocurrent response and electrochemical EIS of the samples.

### 3.3. Photocatalytic Hydrogen Evolution Reaction Tests

The photocatalytic HERs were carried out in a sealed quartz tube reactor with a volume of 250 mL in an online photocatalytic test system (Labsolar-6A, Beijing Perfect Light, Beijing, China). The light source was a 300 W Xe lamp (λ > 400 nm). In a typical test, 25 mg solid catalyst powder was sonically dispersed in a mixture containing 90 mL deionized water and 10 mL TEOA. The reactor was then evacuated and purged with high-purity nitrogen. The photocatalytic reaction was performed at room temperature for 5 h. The H_2_ content was analyzed using an Agilent Micro GC3000 equipped with a molecular sieve 5A column and a high-sensitivity online thermal conductivity detector.

## 4. Conclusions

In conclusion, a novel caviar-like CdS/g-C_3_N_4_ semiconductor nanomaterial was synthesized using a hydrothermal method. This CdS/g-C_3_N_4_ semiconductor exhibited high photocatalytic H_2_ generation activity. XRD patterns and SEM images of pure g-C_3_N_4_ and CdS/g-C_3_N_4_ revealed that doping significantly reduced the agglomeration phase and resulted in the formation of uniformly dispersed caviar-like CdS/g-C_3_N_4_ microspheres with a size of 20–30 nm, indicating a change in the crystal structure and morphology of the nanomaterials. The photoelectrochemical properties and EIS measurements showed that 10 wt% CdS/g-C_3_N_4_ had enhanced absorption properties in the visible light region, an improved transient photocurrent response, and reduced electrochemical impedance after doping. This was potentially due to the introduction of CdS and the formation of a tight interface between the CdS and g-C_3_N_4_ phases. Furthermore, the 10 wt% CdS/g-C_3_N_4_ sample exhibited the highest HER rate of 655.5 μmol/g/h, and this photocatalyst did not exhibit a significant loss of photocatalytic activity across three cycles. The role of CdS nanoparticles in enhancing the photocatalytic HER of g-C_3_N_4_ was significant. In addition to acting as a cocatalyst to accelerate the photocatalytic HER, CdS also enabled the efficient transportation of photogenerated electrons. The dual roles of CdS at the CdS/g-C_3_N_4_ interface were essential for achieving the highest photocatalytic activity. These findings suggest that the integration of CdS/g-C_3_N_4_ nanoheterojunctions could serve as a promising strategy for enhancing the photocatalytic activity of other semiconductor materials for solar energy conversion. Overall, the practical significance of photocatalytic hydrogen evolution lies in its potential to provide a sustainable and renewable source of hydrogen for various industrial and energy-related processes. The high efficiency, low cost, and environmentally friendly nature of photocatalytic HER mean that it is a promising solution for meeting the increasing demand for clean and renewable energy sources. Prospectively, the development of Z-scheme heterojunctions represents a promising avenue for improving the efficiency of solar-light-driven water splitting. While this technology is still in its infancy, ongoing research and development efforts are focused on optimizing the composition and structure of Z-scheme heterojunctions as well as addressing the technical, economic, and environmental challenges associated with their production and implementation. Continued research and development will be necessary to fully harness the full potential of the Z-scheme heterojunctions to drive the transition to a sustainable and renewable energy future.

## Data Availability

Not applicable.
